# Statistical analysis and handling of missing data in cluster randomized trials: a systematic review

**DOI:** 10.1186/s13063-016-1201-z

**Published:** 2016-02-09

**Authors:** Mallorie H. Fiero, Shuang Huang, Eyal Oren, Melanie L. Bell

**Affiliations:** Department of Epidemiology and Biostatistics, Mel and Enid Zuckerman College of Public Health, University of Arizona, 1295 N. Martin Ave., Drachman Hall, P.O. Box 245163, Tucson, Arizona 85724 USA

**Keywords:** Cluster randomized trials, Missing data, Dropout, Sensitivity analysis

## Abstract

**Background:**

Cluster randomized trials (CRTs) randomize participants in groups, rather than as individuals and are key tools used to assess interventions in health research where treatment contamination is likely or if individual randomization is not feasible. Two potential major pitfalls exist regarding CRTs, namely handling missing data and not accounting for clustering in the primary analysis. The aim of this review was to evaluate approaches for handling missing data and statistical analysis with respect to the primary outcome in CRTs.

**Methods:**

We systematically searched for CRTs published between August 2013 and July 2014 using PubMed, Web of Science, and PsycINFO. For each trial, two independent reviewers assessed the extent of the missing data and method(s) used for handling missing data in the primary and sensitivity analyses. We evaluated the primary analysis and determined whether it was at the cluster or individual level.

**Results:**

Of the 86 included CRTs, 80 (93 %) trials reported some missing outcome data. Of those reporting missing data, the median percent of individuals with a missing outcome was 19 % (range 0.5 to 90 %). The most common way to handle missing data in the primary analysis was complete case analysis (44, 55 %), whereas 18 (22 %) used mixed models, six (8 %) used single imputation, four (5 %) used unweighted generalized estimating equations, and two (2 %) used multiple imputation. Fourteen (16 %) trials reported a sensitivity analysis for missing data, but most assumed the same missing data mechanism as in the primary analysis. Overall, 67 (78 %) trials accounted for clustering in the primary analysis.

**Conclusions:**

High rates of missing outcome data are present in the majority of CRTs, yet handling missing data in practice remains suboptimal. Researchers and applied statisticians should carry out appropriate missing data methods, which are valid under plausible assumptions in order to increase statistical power in trials and reduce the possibility of bias. Sensitivity analysis should be performed, with weakened assumptions regarding the missing data mechanism to explore the robustness of results reported in the primary analysis.

**Electronic supplementary material:**

The online version of this article (doi:10.1186/s13063-016-1201-z) contains supplementary material, which is available to authorized users.

## Background

In cluster randomized trials (CRTs), groups of participants, rather than individuals, are randomized to intervention arms. CRTs are often adopted to reduce treatment contamination or if individual randomization is unsuitable and are an increasingly popular approach in comparative effectiveness research [1–4]. In cluster-level allocation, participants cannot be assumed as independent because of the similarity among participants within the same cluster or cluster characteristics, leading to intracluster correlation, or equivalently, between-cluster variation [3]. Two potential pitfalls with respect to CRTs are handling missing data and not accounting for clustering in the primary analysis.

Missing data decreases power and precision and can lead to bias by compromising randomization. For example, treatment arm imbalance with respect to missing data is likely to introduce bias when the outcome is related to the reason for patient withdrawal. Even if missing outcome data are balanced across treatment arms, differing reasons for the missing outcome can cause bias [5]. Reviews of individually randomized controlled trials have discovered that most trials have some missing outcome data [6, 7]. Few reports have discussed missing data in CRTs, despite its high likelihood and the recognition that it poses a serious threat to research validity, as discussed by the National Research Council and the Patient Centered Outcomes Research Institute [5, 8].

Missing data mechanisms are commonly classified into the following three categories. Data are considered to be missing completely at random (MCAR) if missingness is independent of the observed outcomes and covariates. MCAR is a strong assumption and is not likely in most clinical trials. A more sensible assumption is missing at random (MAR), where missingness does not depend on unobserved data after conditioning on the observed data. Data are termed missing not at random (MNAR) if missingness is dependent on unobserved data values even after conditioning on fully observed data [9, 10].

The most common approach for handling missing outcome data is a complete case analysis, which excludes individuals with missing data. This approach yields unbiased estimation if missingness is independent of the outcome, given the covariates [11]. Additional approaches include imputation (single and multiple) and model-based methods. Single imputation strategies, such as the popular last observation carried forward (LOCF) used in longitudinal studies, or mean substitution, replaces missing data with a single number, which underestimates uncertainty [12, 13]. LOCF also makes unlikely assumptions about an individual’s trajectory and can lead to either under- or overestimation of treatment effects [14].

Under the MAR assumption, multiple imputation (MI) considers uncertainty by filling in missing data from a distribution of likely values. Analysis is performed on each dataset and the results combined using specified algorithms. Most implementations of MI are single level, ignoring the multilevel structure of CRTs. Multilevel MI incorporates the lack of independence found within clusters due to the hierarchical data structure found in CRTs [15].

Likelihood based mixed models are valid for MAR data if the model is specified correctly, while unweighted GEE are valid under MCAR if there are a large number of clusters [16, 17]. In order to make a valid complete case analysis under the MAR assumption, inverse probability weighting (IPW) weights complete cases with the inverse of their probability of being observed [18]. Although IPW is relatively simple to perform with monotone missing data, it is prone to large weights, which cause unstable estimates and high variance [10].

The second difficulty regarding CRTs is accounting for clustering in the primary analysis. Ignoring clustering can lead to confidence intervals that are too narrow and increased type I error rates [19, 20]. In order to account for clustering, analysis can be performed at the cluster level or at the individual level. Cluster-level analysis reduces observations within a cluster to an aggregate value and then analyzes each independent data point [20, 21]. Although cluster level analysis alleviates the issue of dependent data, reducing all observations within a cluster to a single summary measure decreases the sample size and power. Analyses at the individual level using general linear models (GLMs) account for non-independent observations within clusters through robust standard errors or adjust using the design effect, an inflation factor used to achieve the same power of an individually randomized trial [22]. Modeling techniques such as generalized estimating equations (GEE) [23] and mixed models [24] explicitly involve intracluster correlation in the modeling process, which enables a more realistic model of the clustered data [24, 25]. Although these models can reduce bias by controlling for confounding at the individual level, they require a higher sample size of a large number of clusters [1, 17, 21].

There have been several reviews on methodological aspects of CRTs (see for example, Simpson et al. [26] and Campbell et al. [27], and the references therein). Diaz-Ordaz et al. [28] reviewed the imputation methods used to handle missing data in CRTs but did not distinguish whether a complete case analysis, GEE, or mixed model was used to handle missing data in the primary analysis, as these approaches provide valid estimates under differing missing data assumptions. Thus, our objective was to provide a comprehensive review of how missing data are being dealt with in CRTs. The primary aims of our review were to accomplish the following:

1. Identify the proportion of CRTs with missing data at the cluster and individual level.
2. Examine the analytical approaches for the primary analysis to find out whether
  - whether missing data had been accommodated and
  - whether clustering had been accounted for.
3. Identify the proportion of CRTs reporting a sensitivity analysis for missing data.

Secondary aims included assessing the techniques for achieving balance in CRTs (stratification, matching, or minimization), the differences between observed and expected attrition rates, and the intracluster correlation.

## Methods

This study was a systematic review of a sample of CRTs published between August 2013 and July 2014. Our methodological strategy was based on guidelines from the Preferred Reporting Items for Systematic Reviews and Meta-Analysis (PRISMA) statement (See Additional file 1 for compliance details) [29]. We have reported a detailed protocol for this study elsewhere [30].

### Eligibility criteria

Eligible studies were restricted to CRTs published in English between August 2013 and July 2014. We included all types of CRTs with human participants, including stepped wedge trials that were reported in the databases listed below [31, 32]. We excluded trial protocols, non- or quasi-experimental designs, secondary trial reports, cost-effectiveness reports, and studies where no individual-level data were collected. We also excluded trials where the primary outcome was survival, as time-to-event analyses handle censored data differently than other types of data.

### Literature search and study selection

Two authors (MF and SH) electronically searched for studies found in PubMed, Web of Science (all databases), and PsycINFO. Titles and abstracts were searched containing the terms “cluster randomized [randomised],” cluster and trial, “community trial,” “community randomized [randomised],” or “group randomized [randomised].” Two independent reviewers (MF and SH) screened titles and abstracts, removed duplicates, and screened full texts.

Both reviewers (MF and SH) and the senior author (MB) performed pilot testing of the data extraction form. All papers used for piloting were included in the systematic review. The reviewers extracted data from each trial using a standardized, pilot-tested form. Disagreements over study eligibility or data extraction were resolved by discussion or with the assistance of a third reviewer (MB) when needed.

### Sample Size

Based on previous literature, it was estimated that about 90 % of trials would report some missing outcome data [6, 7]. Using the formula for a 95 % confidence interval (CI) for a proportion, we estimated that a sample size of 86 papers would result in an acceptable 95 % CI for the hypothesized 90 % of studies having some missing outcome data (95 % CI of 84 to 96).

### Analysis

We defined the number of clusters (and participants) in each trial as the number of clusters (and participants) at randomization. We computed the average number of participants per cluster by dividing the number of participants by the number of clusters.

#### Description and handling of missing data

We evaluated the degree of missing data and the method(s) for handling missing data in the primary analysis for each trial. The primary analysis was defined as the main analysis of the primary outcome. When multiple primary outcomes were reported, we used the first outcome listed in the methods section. For primary outcomes measured repeatedly, we used the final follow-up time point to calculate the missing proportion, unless a different time point was specified for the primary analysis.

The proportion of clusters with a missing outcome was calculated as the number of entire clusters with a missing outcome (generally due to the entire cluster dropping out) divided by the number of clusters randomized. Clusters that were randomized but failed to recruit were considered missing. A similar calculation was carried out for the proportion of participants with a missing outcome. In cases where an entire cluster dropped out, the missing data rate was included in our calculation of missing participants. If the trial had longitudinal data, we calculated the missing rate at the last time point or time point of the primary analysis if specified. Of those who reported some missing data, we identified the statistical methods used to handle missing data, classified into the following categories: complete case, single imputation (such as worst case or LOCF), MI (single level or multilevel), GEE, mixed model or IPW. Technically, mixed models and GEE are considered complete case analyses. However, we make the distinction because these are model-based methods. Mixed models are valid under MAR, and GEE can be modified to be valid under MAR. We also reported methods for missing data for trials indicating greater than or less than 10 % missing data at the individual level. We indicated that a trial presented a sample size calculation if there was enough detail for replication. We recorded whether sample size calculations accounted for missing data, and compared observed and expected attrition rates with the mean absolute difference. If a range was reported for attrition rates, we used the upper bound.

#### Sensitivity analysis for missing data

We computed the number of trials that reported performing a sensitivity analysis and determined the method(s) used to deal with missing data in any sensitivity analysis. Sensitivity analysis was defined as any analysis performed to assess the robustness of the primary results due to changes in assumptions regarding missing outcome data. We also reported methods for sensitivity analysis for trials indicating greater than or less than 10 % missing data at the individual level. We quantified the number of trials that weakened the missingness assumption of their primary analysis (MCAR → MAR → MNAR) to perform their sensitivity analysis as suggested by the Panel on Handling Missing Data in Clinical Trials [10].

#### Accounting for clustering in the primary analysis

For each trial, we calculated the proportion of CRTs performing an individual-level or cluster-level analysis and whether the analysis accounted for clustering. Individual level analyses were categorized into the following groups: basic inferential test (such as *t*-test or chi-square)/GLM (such as linear or logistic regression), GEE, or mixed model. The analysis accounted for clustering if the basic inferential test or GLM obtained robust standard errors or was adjusted using the design effect, if GEE introduced an exchangeable correlation structure for clusters, or if the mixed model used clusters as a random effect. Basic inferential tests/GLMs could also be carried out as a cluster-level analysis. We examined whether the primary analysis was unadjusted, adjusted for baseline variables, adjusted for balance variables such as stratification, or adjusted for additional covariates.

The intracluster correlation coefficient (ICC) measures the degree of similarity among responses within a cluster and is defined as the proportion of total variance due to between-cluster variation. The coefficient of variation (CV) is an alternate measure of between-cluster variability and is defined by the ratio of the standard deviation of cluster sizes to the mean cluster size [3]. We recorded whether trials accounted for clustering in sample size calculations and compared the observed and expected ICCs (or CVs) with the mean absolute difference. If a range was reported for the ICC (or CV), we used the upper bound.

## Results

**Fig. 1:**
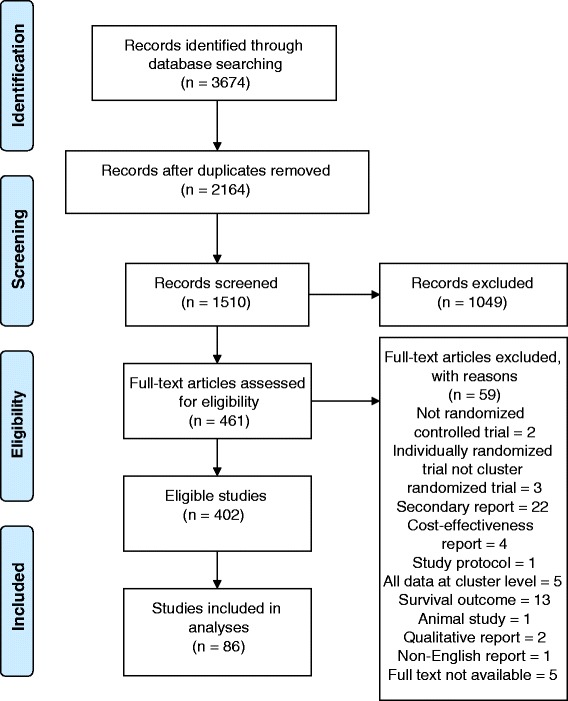
Flow diagram of the study selection process for the sample of 86 cluster randomized trials included in the review

We identified 3,674 records through our electronic database search after removing 2,164 duplicates. We screened 1,510 of the remaining records, of which, 1,049 were excluded, based on titles or abstracts, as not meeting our eligibility criteria. We examined the full texts of the remaining 461 trials and excluded a further 59 trials, as they did not meet eligibility criteria. Of the 402 eligible reports, we used six for piloting and randomly selected 80 others, thereby including 86 trials in the analyses (Fig. 1). The full list of the included studies is given in Additional file 2.

Table 1 presents the general characteristics of the included trials. In total, the median number of clusters randomized was 24, with a range of 2 to 1,552. Three trials were unclear in the number of clusters randomized. The median number of individuals included was 688, with a range of 49 to 117,100. The average number of individuals per cluster ranged from 1 to 1,105. Of the 65 trials that collected the outcome repeatedly, 36 (55 %) used all of the information in the primary analysis by treating the outcome as a repeated measurement, while 29 (45 %) were analyzed at a single time point. Forty-four (51 %) trials used balance techniques to ensure balance after randomization. Stratification was the most common method (27, 61 %), a subset of which also used matching (1) and minimization (1). Fourteen (32 %) of the trials carrying out balance methods used matching, and three (7 %) used minimization.

**Table 1:** General characteristics of the 86 randomly selected cluster randomized trials published from August 2013 to July 2014

	N (%)
Stepped wedge	4 (5)
Pilot/feasibility	4 (5)
Type of outcome	
Quantitative	41 (48)
Binary	37 (43)
Count	8 (9)
How often outcome was collected	
Single	21 (24)
Repeated	65 (76)
How outcome was treated in the primary analysis	
Single	50 (58)
Repeated	36 (42)
Balance methods used in randomization	
Stratification	27 (31)^a^
Matching	14 (16)
Minimization	3 (3)
None	42 (49)
Presented sample size calculation	60 (70)

^a^One trial also used matching, and another trial also used minimization

### Description and handling of missing data

**Fig. 2:**
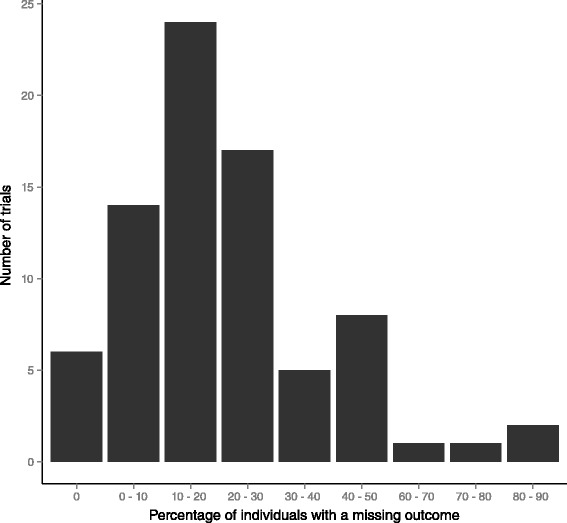
Distribution of the percentage of individuals with a missing outcome for the 86 trials included in the review

Twenty-seven (31 %) trials reported having whole clusters missing in the primary analysis (Table 2). Of these, the median amount of clusters missing was 7 %, with a range of 0.8 to 51 %. Three trials had an unclear number of clusters missing. Reasons for whole clusters missing included closures, natural disasters, a lack of eligible participants, and an inability to retrieve data. Figure 2 displays the proportions of included individuals with missing outcomes. Eighty (93 %) trials reported having some missing data at the individual level. Of these trials, the median amount of missing individual level data was 19 %, with a range of 0.5 to 90 %. Eight trials were unclear in the amount of individual-level missing data. Of the trials reporting some missing data, 61 (76 %) reported reasons for individuals missing, two (2 %) reported missing data due to missing covariates in the adjusted analyses, and 17 (22 %) were unclear or did not report reasons for individuals missing.

**Table 2:** Proportion of clusters with missing outcome at the primary analysis among the 86 trials included in the review

	N (%)
None	59 (69)
<10 %	14 (16)
>10 %	10 (12)
Unclear	3 (3)

The most common approach for handling missing data in the primary analysis was a complete case analysis (44, 55 %) (Table 3). Eighteen (22 %) trials used mixed models. Six (8 %) carried out single imputation methods: three used worst-case imputation, two used LOCF, and one used baseline observation carried forward. Four (5 %) trials used unweighted GEE. Two (2 %) trials performed MI, although neither used multilevel methods. A MAR assumption for the primary analysis was made in 20 (25 %) of the trials with missing data.

**Table 3:** Handling of missing data in primary analysis among the 80 trials who reported missing outcome data

Methods	<10 % missing	>10 % missing	Unclear	Total
	N = 14	N = 58	N = 8	N = 80
Complete case	10	31	3	44 (55)
Single imputation				
Worst-case	1	2	0	3 (4)
LOCF	0	2	0	2 (2)
Baseline observation carried forward	0	1	0	1 (1)
Multiple imputation	0	2	0	2 (2)
GEE (unweighted)	3	0	1	4 (5)
Mixed model/hierarchical/multilevel	0	17	1	18 (22)
Other^a^	0	0	1	1 (1)
Unclear	0	3	2	5 (6)

Abbreviations: LOCF, last observation carried forward; GEE, generalized estimating equation

^a^One trial excluded participants who dropped out or had no baseline value; for those who participated at both time points, the LOCF was carried out for a missing primary outcome

Of the 58 trials reporting more than 10 % missing data at the individual level, 31 (53 %) used complete case analysis, 17 (29 %) used mixed models, five (9 %) used single imputation, two (3 %) used MI, and three (3 %) used methods that were unclear. Of the 14 trials reporting less than 10 % missing data at the individual level, 10 (71 %) used complete case, three (21 %) used unweighted GEE, and one (7 %) used single imputation.

Sixty (70 %) trials presented a sample size calculation, of which 28 (47 %) accounted for missing data via sample size inflation. Twenty-six of these trials accounted for missing data at the individual level, either by dividing by (1 - the estimated dropout rate) or multiplying by (1 + the estimated dropout rate). Two trials also accounted for missing data at the cluster level by including extra clusters in each trial arm. Two trials mentioned sample size inflation but were unclear if they accounted for missing data at the cluster or individual level. Of the 21 trials that reported an expected and observed attrition rate, one trial estimated a higher attrition rate than observed, whereas 20 (95 %) estimated lower attrition rates than observed. The mean absolute difference in observed attrition rate and expected was 9 % with a range of 0.1 to 23 %.

### Sensitivity analysis for missing data

Fourteen (16 %) trials reported a sensitivity analysis for missing data (Table 4), all of which reported more than 10 % missing data at the individual level. Of these, five (36 %) used MI (none of which used multilevel strategies), four (29 %) used single imputation, three (21 %) used a complete case analysis, one (7 %) used a mixed model, and one (7 %) used a mixed model with IPW.

**Table 4:** Methods for handling missing data in sensitivity analysis in 14 trials

Sensitivity method	Primary analysis	N	Total N (%)
Complete case	MI	2	3 (21)
	Mixed model	1	
Single imputation	Complete case	1	4 (29)
	Single imputation	1	
	Mixed model	2	
MI	Complete case	3	5 (36)
	Mixed model	1	
	Unclear	1	
Mixed model	Complete case	1	1 (7)
Mixed model with IPW	Complete case	1	1 (7)

Abbreviations: MI, multiple imputation; IPW, inverse probability weighting

Only five trials weakened the missingness assumption of the primary analysis to carry out their sensitivity analysis by assuming MCAR in the primary analysis and MAR in the sensitivity analysis. These five trials all used a complete case analysis as the primary analysis. For the sensitivity analysis, three of these trials used MI, one used a mixed model, and one used a mixed model with IPW. None of the trials reported using MNAR models.

### Accounting for clustering in the primary analysis

The overwhelming majority of trials carried out an individual-level analysis as the primary analysis (83, 97 %). Mixed models were the most popular primary analysis used for CRTs (45, 52 %). Forty-three (96 %) of these trials accounted for clustering by adding cluster as a random effect, one trial was unclear, and one did not use cluster as a random effect. Of the 22 (26 %) trials performing an individual level basic inferential test or GLM, seven accounted for clustering via robust standard errors or design effect adjustment. Fourteen (16 %) trials used GEE, with all of them accounting for clustering by using an exchangeable correlation structure. Of these, one reported estimating standard errors of parameters using the jack-knife method because the number of clusters was small [33]. One (1 %) trial carried out a descriptive analysis as the primary analysis and did not account for clustering (Table 5). Four (5 %) trials carried out a basic inferential test or GLM at the cluster level. Overall, 68 (79 %) trials accounted for clustering in the primary analysis.

**Table 5:** Primary analysis in 86 cluster randomized trials

	Accounted for clustering^a^		Total
Primary Analysis	Yes	No	N (%)
	N (%)	N (%)	
Individual level:			
Basic inferential test/GLM	7 (32)	15 (68)	22 (26)
GEE	14 (100)	0 (0)	14 (16)
Mixed model	43 (96)	2 (4)^b^	45 (52)
Other^c^	0 (0)	1 (100)	1 (1)
Cluster level:			
Basic inferential test/GLM	4 (100)	0 (0)	4 (5)

Abbreviations: GLM, generalized linear model; GEE, generalized estimating equation

^a^The denominator is the total number of trials performing respective primary analysis

^b^One trial was unclear

^c^Trial used a descriptive analysis as primary analysis

Thirty-four (40 %) trials carried out an unadjusted analysis, whereas five (6 %) adjusted for balance variables only (stratification, matching, or minimization), and eight (9 %) adjusted for baseline outcome only (sometimes referred to as analysis of covariance (ANCOVA)). Thirty-nine (45 %) trials adjusted for additional covariates beyond balance variables in the primary analysis, with four of them also adjusting for baseline values of the outcome.

Forty-six (77 %) trials reported accounting for clustering in their sample size calculations, with 41 reporting an expected ICC or CV (two trials). Of the 13 trials that reported an expected and observed ICC, seven (54 %) trials estimated larger ICCs than observed, whereas six (46 %) estimated lower ICCs than observed. The mean absolute difference in the observed and expected ICC was 0.1, with a range of 0.01 to 0.42.

## Discussion

We performed a systematic review to assess how missing outcome data are being handled in CRTs. Of the 86 included CRTs, most reported some missing outcome data in the primary analysis. Among those that reported missing data, the median proportion of individuals with a missing outcome at the primary analysis was 19 %. Sixteen percent of the trials carried out a sensitivity analysis for missing data, with all of them reporting more than 10 % missing data. Only a third of these trials weakened the missingness assumption from the primary analysis.

Observed missing data rates generally exceeded expected rates, which means that researchers are not accounting enough for attrition in sample size calculations or adequately following up on participants. Furthermore, only about half (55 %) of the trials with repeated measurements used all of the outcome data in the primary analysis. Reducing repeated data to a single time point often generates a strong MCAR assumption and may reduce power. Even if the primary outcome of interest is at a particular time point, previous literature has shown that utilizing all of the information collected can minimize bias due to missing data [34].

The amount of detail in sample size calculations varied widely across trials. A few did not provide enough detail for us to indicate that a sample size calculation was performed before data collection. For example, one trial stated “sample size calculations showed 382 participants were needed.” [35] Furthermore, accounting for clustered data in sample size calculations differed among trials. One trial arbitrarily chose to increase the sample size by 30 % to account for clustering [36]. One trial stated that clustering was not accounted for in the sample size calculation because cluster sizes were expected to be small and within-cluster comparisons were not considered to be clinically meaningful [37].

Along with missing individuals, missing data can also occur at the cluster level. The removal of entire clusters with the usual solution of complete case analysis is wasteful and could lead to biased estimates depending on the missing data mechanism [38]. We did not find any studies that performed MI appropriate for clustered data (multilevel MI). Some strategies that have been proposed to accommodate missing data in the multilevel setting, but none have been put to widespread use [15, 39–41].

In comparison to Diaz-Ordaz et al.’s [28] review, we found a higher proportion of trials reporting missing data at the cluster (28 % versus 18 %) and individual levels (93 % versus 48 %). This may be due to differences in definitions of missing data or because Diaz-Ordaz was not able to verify the amount of missing data in 31 % of the trials. We observed a similar median cluster attrition rate (7 % versus 10 %) and a slightly higher median individual attrition rate (19 % versus 13 %). Of the 95 trials with missing data, Diaz-Ordaz et al. found 66 % of the trials reporting a complete case analysis, GEE, or likelihood-based hierarchical/mixed model, whereas 18 % used single imputation and 6 % used MI. Lastly, we found a slightly higher proportion of trials reporting a sensitivity analysis for missing data (16 % versus 11 %). Compared to Bell et al.’s [7] review of 77 individually randomized controlled trials from 2013, we found a similar proportion of trials reporting missing data (93 % versus 95 %). However, CRTs were subject to higher individual level missing data rates (median 19 %, up to 90 %) compared to individually randomized trials (median 9 %, up to 70 %). Compared to the individually randomized trials, we found a higher proportion using complete case analysis (55 % versus 45 %) and mixed models (22 % versus 15 %). Furthermore, we found a similar proportion using GEE (4 % versus 5 %) and a lower proportion using single imputation (8 % versus 27 %) and MI (2 % versus 8 %)

More sophisticated methods are being used. Compared to a review conducted by Simpson et al. [26] of 21 CRTs from 1990 to 1993, the proportion of trials that took clustering into account in the primary analysis increased over time (57 % to 78 %). In comparison with Scott et al.’s [42] review of 150 individually randomized trials in 2001, we found a higher percentage of CRTs using stratification (31 % versus 13 %) and a similar percentage using minimization (3 % versus 4 %) compared to individually randomized trials.

Our study has several strengths. Eligible studies were all CRT designs, including the stepped wedge and feasibility studies. In order to minimize the potential for bias during the review process, we had pre-specified search, study selection, and data collection strategies, all of which were carried out by two independent reviewers. We did not limit our sample space to journals with a high impact factor, thereby increasing generalizability. Three independent reviewers performed pilot testing on several trials to create a standardized data collection template. Our study has limitations as well. For example, we only chose CRTs published in English, which may result in selection bias. It was difficult to identify all CRTs because many do not include “cluster” as a term in the title or abstract. However, our search strategy included other frequently used terms for cluster randomization such as “community randomized” and “group randomized.” Still, our review may have some selection bias, as researchers who do not realize their studies are cluster randomized might not follow the CONSORT guidelines, include terms such as “cluster randomized” in the title or abstract, or use robust techniques [27]. Additionally, we took a random selection of the eligible CRTs, as it was not feasible to review all 402 studies. As with any sample, this one may not be representative of the true population. However, a random selection minimizes the possibility of non-representativeness. Furthermore, we may have underestimated the amount of missing data because we used the CONSORT flow diagram, which may primarily report outcome sample size only. It is possible that missing covariates in regression models resulted in additional missing data and actual smaller sample sizes. Although some trials adjusted for additional covariates beyond balance variables, nearly all were baseline covariates such as age and gender.

In conclusion, missing data are present in the majority of CRTs, yet handling missing data in practice remains suboptimal. Appropriate methods to handle missing clustered data, particularly under the MAR assumption, should be made more accessible by methodological statisticians. For example, providing appropriate software may increase the use of such methods [43]. Moreover, researchers and applied statisticians should keep up-to-date with such methods in order to increase statistical power in trials and reduce the potential for bias. Thus, we present the following recommendations for CRTs: (1) attempt to follow up on all randomized clusters and individuals in order to limit the extent of missing data, (2) perform a primary analysis that is valid under a plausible missingness assumption and that uses all observed data, (3) perform sensitivity analyses that weaken the missing data assumption to explore the impact of departures made in the primary analysis, and (4) follow the CONSORT extension for cluster trials statement to ensure comprehensive reporting and transparency of methods [10, 44].

## Conclusions

This review aims to assess the extent and handling of missing outcome data in CRTs. Despite high rates of missing outcome data in the primary analysis, methods used to deal with missing data in practice remain inadequate. Appropriate methods, which are valid under probable missing data assumptions, should be performed to increase the statistical power and lessen the likelihood of bias. Sensitivity analysis with a weakened missing data assumption should be performed to evaluate robustness of the primary results.

## Additional files

Additional file 1:
**PRISMA 2009 Checklist.** (PDF 115 kb) Additional file 2:
**References of the 86 trials included in the review.** (PDF 90 kb)

## Abbreviations

CI: confidence interval
CV: coefficient of variation
GEE: generalized estimating equation
GLM: generalized linear model
ICC: intracluster correlation coefficient
IPW: inverse probability weighting
MAR: missing at random
MCAR: missing completely at random
MI: multiple imputation
MNAR: missing not at random
