# Supplementary material for: Statistical analysis and handling of missing data in cluster randomized trials: a systematic review
Source: Trials. 2016 Feb 9;17:72. doi: 10.1186/s13063-016-1201-z (PMC4748550; doi:10.1186/s13063-016-1201-z)
Supplement: Additional file 2: — References of the 86 trials included in the review. (PDF 90 kb) [file 13063_2016_1201_MOESM2_ESM.pdf]

Supplemental File

Below are the references of the 86 trials included in the review.

1. Arvidsson H, Olin E, Strand J, et al. Effects of the two-way communication checklist (2-COM): a one-year cluster randomized study in a group of severely mentally ill persons. Int J Soc Psychiatry 2014;**60**(1):95-102 doi: 10.1177/0020764012467145.
2. Baatjies R, Meijster T, Heederik D, et al. Effectiveness of interventions to reduce flour dust exposures in supermarket bakeries in South Africa. Occup Environ Med 2014;**71**(12):811-8 doi: 10.1136/oemed-2013-101971.
3. Bavarian N, Lewis KM, Dubois DL, et al. Using social-emotional and character development to improve academic outcomes: a matched-pair, cluster-randomized controlled trial in low-income, urban schools. J Sch Health 2013;**83**(11):771-9 doi: 10.1111/josh.12093.
4. Bird C, Ame S, Albonico M, et al. Do shoes reduce hookworm infection in school-aged children on Pemba Island, Zanzibar? A pragmatic trial. Trans R Soc Trop Med Hyg 2014;**108**(5):297-304 doi: 10.1093/trstmh/tru037.
5. Campbell L, Novak I, McIntyre S, et al. A KT intervention including the evidence alert system to improve clinician's evidence-based practice behavior--a cluster randomized controlled trial. Implement Sci 2013;**8**:132 doi: 10.1186/1748-5908-8-132.
6. Carroll AE, Bauer NS, Dugan TM, et al. Use of a computerized decision aid for developmental surveillance and screening: a randomized clinical trial. JAMA Pediatr 2014;**168**(9):815-21 doi: 10.1001/jamapediatrics.2014.464.
7. Chan SS, Leung DY, Leung AY, et al. A nurse-delivered brief health education intervention to improve pneumococcal vaccination rate among older patients with chronic diseases: A cluster randomized controlled trial. Int J Nurs Stud 2015;**52**(1):317-24 doi: 10.1016/j.ijnurstu.2014.06.008.
8. Cohen KE, Morgan PJ, Plotnikoff RC, et al. Physical Activity and Skills Intervention: SCORES Cluster Randomized Controlled Trial. Med Sci Sports Exerc 2014 doi: 10.1249/MSS.0000000000000452.
9. Colon-Emeric CS, McConnell E, Pinheiro SO, et al. CONNECT for better fall prevention in nursing homes: results from a pilot intervention study. J Am Geriatr Soc 2013;**61**(12):2150-9 doi: 10.1111/jgs.12550.
10. Connor CM, Morrison FJ, Fishman B, et al. A longitudinal cluster-randomized controlled study on the accumulating effects of individualized literacy instruction on students' reading from first through third grade. Psychol Sci 2013;**24**(8):1408-19 doi: 10.1177/0956797612472204.
11. Craig T, Shepherd G, Rinaldi M, et al. Vocational rehabilitation in early psychosis: cluster randomised trial. Br J Psychiatry 2014;**205**(2):145-50 doi: 10.1192/bjp.bp.113.136283.
12. de Graaff JC, Cuper NJ, Mungra RA, et al. Near-infrared light to aid peripheral intravenous cannulation in children: a cluster randomised clinical trial of three devices. Anaesthesia 2013;**68**(8):835-45 doi: 10.1111/anae.12294.
13. Deales A, Fratini M, Romano S, et al. Care manager to control cardiovascular risk factors in primary care: the Raffaello cluster randomized trial. Nutr Metab Cardiovasc Dis 2014;**24**(5):563-71 doi: 10.1016/j.numecd.2013.11.008.
14. Deressa W, Yihdego YY, Kebede Z, et al. Effect of combining mosquito repellent and insecticide treated net on malaria prevalence in Southern Ethiopia: a cluster-randomised trial. Parasit Vectors 2014;**7**:132 doi: 10.1186/1756-3305-7-132.

15. Dixon A, Clarkin C, Barrowman N, et al. Reduction of radial-head subluxation in children by triage nurses in the emergency department: a cluster-randomized controlled trial. CMAJ 2014;**186**(9):E317-23 doi: 10.1503/cmaj.131101.
16. Duda JL, Williams GC, Ntoumanis N, et al. Effects of a standard provision versus an autonomy supportive exercise referral programme on physical activity, quality of life and well-being indicators: a cluster randomised controlled trial. Int J Behav Nutr Phys Act 2014;**11**:10 doi: 10.1186/1479-5868-11-10.
17. Ebenezer R, Gunawardena K, Kumarendran B, et al. Cluster-randomised trial of the impact of school-based deworming and iron supplementation on the cognitive abilities of schoolchildren in Sri Lanka's plantation sector. Trop Med Int Health 2013;**18**(8):942-51 doi: 10.1111/tmi.12128.
18. Fink G, Robyn PJ, Sié A, et al. Does health insurance improve health?: Evidence from a randomized community-based insurance rollout in rural Burkina Faso. J Health Econ 2013;**32**(6):1043-56 doi: 10.1016/j.jhealeco.2013.08.003.
19. Flax VL, Negerie M, Ibrahim AU, et al. Integrating group counseling, cell phone messaging, and participant-generated songs and dramas into a microcredit program increases Nigerian women's adherence to international breastfeeding recommendations. J Nutr 2014;**144**(7):1120-4 doi: 10.3945/jn.113.190124.
20. Freiberger E, Blank WA, Salb J, et al. Effects of a complex intervention on fall risk in the general practitioner setting: a cluster randomized controlled trial. Clin Interv Aging 2013;**8**:1079-88 doi: 10.2147/CIA.S46218.
21. Fuller JM, Wong KK, Grunstein R, et al. A comparison of screening methods for sleep disorders in Australian community pharmacies: a randomized controlled trial. PLoS One 2014;**9**(6):e101003 doi: 10.1371/journal.pone.0101003.
22. Galik E, Resnick B, Hammersla M, et al. Optimizing function and physical activity among nursing home residents with dementia: testing the impact of function-focused care. Gerontologist 2014;**54**(6):930-43 doi: 10.1093/geront/gnt108.
23. Gärtner FR, Nieuwenhuijsen K, Ketelaar SM, et al. The mental vitality @ work study: effectiveness of a mental module for workers' health surveillance for nurses and allied health care professionals on their help-seeking behavior. J Occup Environ Med 2013;**55**(10):1219-29 doi: 10.1097/JOM.0b013e31829f310a.
24. Haller DM, Meynard A, Lefebvre D, et al. Effectiveness of training family physicians to deliver a brief intervention to address excessive substance use among young patients: a cluster randomized controlled trial. CMAJ 2014;**186**(8):E263-72 doi: 10.1503/cmaj.131301.
25. Hamid S, Dunsiger S, Seiden A, et al. Impact of a diabetes control and management intervention on health care utilization in American Samoa. Chronic Illn 2014;**10**(2):122-34 doi: 10.1177/1742395313502367.
26. Haugen AS, Søfteland E, Almeland SK, et al. Effect of the World Health Organization Checklist on Patient Outcomes: A Stepped Wedge Cluster Randomized Controlled Trial. Ann Surg 2014 doi: 10.1097/SLA.0000000000000716.
27. Heyland DK, Murch L, Cahill N, et al. Enhanced protein-energy provision via the enteral route feeding protocol in critically ill patients: results of a cluster randomized trial. Crit Care Med 2013;**41**(12):2743-53 doi: 10.1097/CCM.0b013e31829efef5.
28. Hiemstra M, Ringlever L, Otten R, et al. Long-term effects of a home-based smoking prevention program on smoking initiation: a cluster randomized controlled trial. Prev Med 2014;**60**:65-70 doi: 10.1016/j.ypmed.2013.12.012.
29. Hirani SP, Beynon M, Cartwright M, et al. The effect of telecare on the quality of life and psychological well-

being of elderly recipients of social care over a 12-month period: the Whole Systems Demonstrator cluster randomised trial. Age Ageing 2014;**43**(3):334-41 doi: 10.1093/ageing/aft185.
30. Inauen J, Tobias R, Mosler HJ. The role of commitment strength in enhancing safe water consumption: mediation analysis of a cluster-randomized trial. Br J Health Psychol 2014;**19**(4):701-19 doi: 10.1111/bjhp.12068.
31. Isensee B, Hansen J, Maruska K, et al. Effects of a school-based prevention programme on smoking in early adolescence: a 6-month follow-up of the 'Eigenstandig werden' cluster randomised trial. BMJ Open 2014;**4**(1):e004422 doi: 10.1136/bmjopen-2013-004422.
32. Ismail KM, Kettle C, Macdonald SE, et al. Perineal Assessment and Repair Longitudinal Study (PEARLS): a matched-pair cluster randomized trial. BMC Med 2013;**11**:209 doi: 10.1186/1741-7015-11-209.
33. Kauye F, Jenkins R, Rahman A. Training primary health care workers in mental health and its impact on diagnoses of common mental disorders in primary care of a developing country, Malawi: a cluster-randomized controlled trial. Psychol Med 2014;**44**(3):657-66 doi: 10.1017/S0033291713001141.
34. Ketelaar SM, Nieuwenhuijsen K, Gärtner FR, et al. Effect of an E-mental health approach to workers' health surveillance versus control group on work functioning of hospital employees: a cluster-RCT. PLoS One 2013;**8**(9):e72546 doi: 10.1371/journal.pone.0072546.
35. Lerner-Geva L, Bar-Zvi E, Levitan G, et al. An intervention for improving the lifestyle habits of kindergarten children in Israel: a cluster-randomised controlled trial investigation. Public Health Nutr 2014:1-8 doi: 10.1017/S136898001400024X.
36. Little P, Stuart B, Francis N, et al. Effects of internet-based training on antibiotic prescribing rates for acute respiratory-tract infections: a multinational, cluster, randomised, factorial, controlled trial. Lancet 2013;**382**(9899):1175-82 doi: 10.1016/S0140-6736(13)60994-0.
37. Madigan SM, Fleming P, Wright ME, et al. A cluster randomised controlled trial of a nutrition education intervention in the community. J Hum Nutr Diet 2014;**27 Suppl 2**:12-20 doi: 10.1111/jhn.12079.
38. McCrow J, Sullivan KA, Beattie ER. Delirium knowledge and recognition: a randomized controlled trial of a web-based educational intervention for acute care nurses. Nurse Educ Today 2014;**34**(6):912-7 doi: 10.1016/j.nedt.2013.12.006.
39. Meeks S, Van Haitsma K, Schoenbachler B, et al. BE-ACTIV for Depression in Nursing Homes: Primary Outcomes of a Randomized Clinical Trial. J Gerontol B Psychol Sci Soc Sci 2015;**70**(1):13-23 doi: 10.1093/geronb/gbu026.
40. Menchetti M, Sighinolfi C, Di Michele V, et al. Effectiveness of collaborative care for depression in Italy. A randomized controlled trial. Gen Hosp Psychiatry 2013;**35**(6):579-86 doi: 10.1016/j.genhosppsych.2013.07.009.
41. Mengistie B, Berhane Y, Worku A. Household water chlorination reduces incidence of diarrhea among under-five children in rural Ethiopia: a cluster randomized controlled trial. PLoS One 2013;**8**(10):e77887 doi: 10.1371/journal.pone.0077887.
42. Meyer U, Schindler C, Zahner L, et al. Long-term effect of a school-based physical activity program (KISS) on fitness and adiposity in children: a cluster-randomized controlled trial. PLoS One 2014;**9**(2):e87929 doi: 10.1371/journal.pone.0087929.
43. Muhumuza S, Olsen A, Katahoire A, et al. Effectiveness of a pre-treatment snack on the uptake of mass treatment for schistosomiasis in Uganda: a cluster randomized trial. PLoS Med 2014;**11**(5):e1001640 doi: 10.1371/journal.pmed.1001640.

44. Na JU, Lee TR, Kang MJ, et al. Basic life support skill improvement with newly designed renewal programme: cluster randomised study of small-group-discussion method versus practice-while-watching method. Emerg Med J 2014;**31**(12):964-9 doi: 10.1136/emermed-2013-202379.
45. Nauta J, Knol DL, Adriaensens L, et al. Prevention of fall-related injuries in 7-year-old to 12-year-old children: a cluster randomised controlled trial. Br J Sports Med 2013;**47**(14):909-13 doi: 10.1136/bjsports-2012-091439.
46. Ochola SA, Labadarios D, Nduati RW. Impact of counselling on exclusive breast-feeding practices in a poor urban setting in Kenya: a randomized controlled trial. Public Health Nutr 2013;**16**(10):1732-40 doi: 10.1017/S1368980012004405.
47. Palmu AA, Jokinen J, Nieminen H, et al. Effect of pneumococcal Haemophilus influenzae protein D conjugate vaccine (PHiD-CV10) on outpatient antimicrobial purchases: a double-blind, cluster randomised phase 3-4 trial. Lancet Infect Dis 2014;**14**(3):205-12 doi: 10.1016/S1473-3099(13)70338-4.
48. Papish A, Kassam A, Modgill G, et al. Reducing the stigma of mental illness in undergraduate medical education: a randomized controlled trial. BMC Med Educ 2013;**13**:141 doi: 10.1186/1472-6920-13-141.
49. Pasha O, McClure EM, Wright LL, et al. A combined community- and facility-based approach to improve pregnancy outcomes in low-resource settings: a Global Network cluster randomized trial. BMC Med 2013;**11**:215 doi: 10.1186/1741-7015-11-215.
50. Penfold S, Manzi F, Mkumbo E, et al. Effect of home-based counselling on newborn care practices in southern Tanzania one year after implementation: a cluster-randomised controlled trial. BMC Pediatr 2014;**14**:187 doi: 10.1186/1471-2431-14-187.
51. Power M, Tyrrell PJ, Rudd AG, et al. Did a quality improvement collaborative make stroke care better? A cluster randomized trial. Implement Sci 2014;**9**(1):40 doi: 10.1186/1748-5908-9-40.
52. Primack BA, Douglas EL, Land SR, et al. Comparison of media literacy and usual education to prevent tobacco use: a cluster-randomized trial. J Sch Health 2014;**84**(2):106-15 doi: 10.1111/josh.12130.
53. Rat C, Quereux G, Riviere C, et al. Targeted melanoma prevention intervention: a cluster randomized controlled trial. Ann Fam Med 2014;**12**(1):21-8 doi: 10.1370/afm.1600.
54. Reynolds GS, Bennett JB. A cluster randomized trial of alcohol prevention in small businesses: a cascade model of help seeking and risk reduction. Am J Health Promot 2015;**29**(3):182-91 doi: 10.4278/ajhp.121212-QUAN-600.
55. Richards DA, Hill JJ, Gask L, et al. Clinical effectiveness of collaborative care for depression in UK primary care (CADET): cluster randomised controlled trial. BMJ 2013;**347**:f4913
56. Richter L, Rotheram-Borus MJ, Van Heerden A, et al. Pregnant women living with HIV (WLH) supported at clinics by peer WLH: a cluster randomized controlled trial. AIDS Behav 2014;**18**(4):706-15 doi: 10.1007/s10461-014-0694-2.
57. Saboori S, Greene LE, Moe CL, et al. Impact of regular soap provision to primary schools on hand washing and E. coli hand contamination among pupils in Nyanza Province, Kenya: a cluster-randomized trial. Am J Trop Med Hyg 2013;**89**(4):698-708 doi: 10.4269/ajtmh.12-0387.
58. Santos RG, Durksen A, Rabbanni R, et al. Effectiveness of peer-based healthy living lesson plans on anthropometric measures and physical activity in elementary school students: a cluster randomized trial. JAMA Pediatr 2014;**168**(4):330-7 doi: 10.1001/jamapediatrics.2013.3688.
59. Shakeshaft A, Doran C, Petrie D, et al. The effectiveness of community action in reducing risky alcohol consumption and harm: a cluster randomised controlled trial. PLoS Med 2014;**11**(3):e1001617 doi:

10.1371/journal.pmed.1001617.

60. Smidth M, Olesen F, Fenger-Grøn M, et al. Patient-experienced effect of an active implementation of a disease management programme for COPD - a randomised trial. BMC Fam Pract 2013;**14**:147 doi: 10.1186/1471-2296-14-147.
61. Snow PC, Eadie PA, Connell J, et al. Oral language supports early literacy: a pilot cluster randomized trial in disadvantaged schools. Int J Speech Lang Pathol 2014;**16**(5):495-506 doi: 10.3109/17549507.2013.845691.
62. Sorensen G, Pednekar MS, Sinha DN, et al. Effects of a tobacco control intervention for teachers in India: results of the Bihar school teachers study. Am J Public Health 2013;**103**(11):2035-40 doi: 10.2105/AJPH.2013.301303.
63. Stallard P, Phillips R, Montgomery AA, et al. A cluster randomised controlled trial to determine the clinical effectiveness and cost-effectiveness of classroom-based cognitive-behavioural therapy (CBT) in reducing symptoms of depression in high-risk adolescents. Health Technol Assess 2013;**17**(47):vii-xvii, 1-109 doi: 10.3310/hta17470.
64. Stanton CK, Newton S, Mullany LC, et al. Effect on postpartum hemorrhage of prophylactic oxytocin (10 IU) by injection by community health officers in Ghana: a community-based, cluster-randomized trial. PLoS Med 2013;**10**(10):e1001524 doi: 10.1371/journal.pmed.1001524.
65. Svarstad BL, Kotchen JM, Shireman TI, et al. Improving refill adherence and hypertension control in black patients: Wisconsin TEAM trial. J Am Pharm Assoc (2003) 2013;**53**(5):520-9 doi: 10.1331/JAPhA.2013.12246.
66. Taddio A, Smart S, Sheedy M, et al. Impact of prenatal education on maternal utilization of analgesic interventions at future infant vaccinations: a cluster randomized trial. Pain 2014;**155**(7):1288-92 doi: 10.1016/j.pain.2014.03.024.
67. Tannenbaum C, Agnew R, Benedetti A, et al. Effectiveness of continence promotion for older women via community organisations: a cluster randomised trial. BMJ Open 2013;**3**(12):e004135 doi: 10.1136/bmjopen-2013-004135.
68. Tannenbaum C, Martin P, Tamblyn R, et al. Reduction of inappropriate benzodiazepine prescriptions among older adults through direct patient education: the EMPOWER cluster randomized trial. JAMA Intern Med 2014;**174**(6):890-8 doi: 10.1001/jamainternmed.2014.949.
69. Tine RC, Ndour CT, Faye B, et al. Feasibility, safety and effectiveness of combining home based malaria management and seasonal malaria chemoprevention in children less than 10 years in Senegal: a cluster-randomised trial. Trans R Soc Trop Med Hyg 2014;**108**(1):13-21 doi: 10.1093/trstmh/trt103.
70. Totsu S, Yamasaki C, Terahara M, et al. Bifidobacterium and enteral feeding in preterm infants: cluster-randomized trial. Pediatr Int 2014;**56**(5):714-9 doi: 10.1111/ped.12330.
71. Tran KP, Nguyen Q, Truong XN, et al. A comparison of ketamine and morphine analgesia in prehospital trauma care: a cluster randomized clinical trial in rural Quang Tri province, Vietnam. Prehosp Emerg Care 2014;**18**(2):257-64 doi: 10.3109/10903127.2013.851307.
72. Trost SG, Sundal D, Foster GD, et al. Effects of a pediatric weight management program with and without active video games a randomized trial. JAMA Pediatr 2014;**168**(5):407-13 doi: 10.1001/jamapediatrics.2013.3436.
73. Umanodan R, Shimazu A, Minami M, et al. Evaluation of a Computer-based Stress Management Training Program for Workers' Psychological Well-being and Work Performance: A Cluster Randomized

Controlled Trial. Industrial health 2014
74. Valve P, Lehtinen-Jacks S, Eriksson T, et al. LINDA - a solution-focused low-intensity intervention aimed at improving health behaviors of young females: a cluster-randomized controlled trial. BMC Public Health 2013;**13**:1044 doi: 10.1186/1471-2458-13-1044.
75. van de Steeg L, IJkema R, Langelaan M, et al. Can an e-learning course improve nursing care for older people at risk of delirium: a stepped wedge cluster randomised trial. BMC Geriatr 2014;**14**:69 doi: 10.1186/1471-2318-14-69.
76. Van den Donk M, Griffin SJ, Stellato RK, et al. Effect of early intensive multifactorial therapy compared with routine care on self-reported health status, general well-being, diabetes-specific quality of life and treatment satisfaction in screen-detected type 2 diabetes mellitus patients (ADDITION-Europe): a cluster-randomised trial. Diabetologia 2013 doi: 10.1007/s00125-013-3011-0.
77. Vicens C, Bejarano F, Sempere E, et al. Comparative efficacy of two interventions to discontinue long-term benzodiazepine use: cluster randomised controlled trial in primary care. Br J Psychiatry 2014;**204**(6):471-9 doi: 10.1192/bjp.bp.113.134650.
78. Williams AE, Stevens VJ, Albright CL, et al. The results of a 2-year randomized trial of a worksite weight management intervention. Am J Health Promot 2014;**28**(5):336-9 doi: 10.4278/ajhp.100127-ARB-29.
79. Williams SE, Rothman RL, Offit PA, et al. A randomized trial to increase acceptance of childhood vaccines by vaccine-hesitant parents: a pilot study. Acad Pediatr 2013;**13**(5):475-80 doi: 10.1016/j.acap.2013.03.011.
80. Wilson A, O'Hare JP, Hardy A, et al. Evaluation of the clinical and cost effectiveness of intermediate care clinics for diabetes (ICCD): a multicentre cluster randomised controlled trial. PLoS One 2014;**9**(4):e93964 doi: 10.1371/journal.pone.0093964.
81. Wilson GB, Wray C, McGovern R, et al. Intervention to reduce excessive alcohol consumption and improve comorbidity outcomes in hypertensive or depressed primary care patients: two parallel cluster randomized feasibility trials. Trials 2014;**15**:235 doi: 10.1186/1745-6215-15-235.
82. Wolfenden L, Wyse R, Campbell E, et al. Randomized controlled trial of a telephone-based intervention for child fruit and vegetable intake: long-term follow-up. Am J Clin Nutr 2014;**99**(3):543-50 doi: 10.3945/ajcn.113.071738.
83. Wüsthoff LE, Waal H, Gråwe RW. The effectiveness of integrated treatment in patients with substance use disorders co-occurring with anxiety and/or depression--a group randomized trial. BMC Psychiatry 2014;**14**:67 doi: 10.1186/1471-244X-14-67.
84. Zatzick D, Donovan DM, Jurkovich G, et al. Disseminating alcohol screening and brief intervention at trauma centers: a policy-relevant cluster randomized effectiveness trial. Addiction 2014;**109**(5):754-65 doi: 10.1111/add.12492.
85. Zheng Y, Li XG, Wang QZ, et al. Enhancement of vitamin A combined vitamin D supplementation on immune response to Bacille Calmette-Guérin vaccine revaccinated in Chinese infants. Asian Pac J Trop Med 2014;**7**(2):130-5 doi: 10.1016/S1995-7645(14)60008-0.
86. Zlotkin S, Newton S, Aimone AM, et al. Effect of iron fortification on malaria incidence in infants and young children in Ghana: a randomized trial. JAMA 2013;**310**(9):938-47 doi: 10.1001/jama.2013.277129.
